# Mechanical Ventilator Liberation of Patients With COVID-19 in Long-term Acute Care Hospital

**DOI:** 10.1016/j.chest.2022.02.030

**Published:** 2022-02-25

**Authors:** Tamas Dolinay, Dale Jun, Lucia Chen, Jeffrey Gornbein

**Affiliations:** a Department of Medicine, University of California, Los Angeles, Los Angeles, CA; b Barlow Respiratory Hospital, University of California, Los Angeles, Los Angeles, CA

**Keywords:** COVID-19, long-term acute care hospital, ventilator liberation, BRH, Barlow Respiratory Hospital, CKD, chronic kidney disease, FSS-ICU, Functional Status Score for the Intensive Care Unit, IQR, interquartile range, LOS, length of stay, LTACH, long-term acute care hospital, MV, mechanical ventilation, STACH, short-term acute care hospital

## Abstract

**Background:**

Mechanical ventilation (MV) via tracheostomy is performed commonly for patients who are in long-term acute care hospitals (LTACHs) after respiratory failure. However, the outcome of MV in COVID-19-associated respiratory failure in LTACHs is not known.

**Research Question:**

What is the ventilator liberation rate of patients who have received tracheostomy with COVID-19-associated respiratory failure compared with those with respiratory failure unrelated to COVID-19 in LTACHs?

**Study Design and Methods:**

In this retrospective cohort study, we examined mechanically ventilated patients discharged between June 2020 and March 2021. Of 242 discharges, 165 patients who had undergone tracheostomy arrived and were considered for ventilator liberation. One hundred twenty-eight patients did not have COVID-19 and 37 patients were admitted for COVID-19.

**Results:**

The primary outcome of the study was ventilator liberation; secondary outcomes were functional recovery, length of stay (LOS) at the LTACH, and discharge disposition. After controlling for demographics, the number of comorbidities, hemodialysis, vasopressor need, thrombocytopenia, and the LOS at the short-term acute care hospital, our results indicated that patients with COVID-19 showed a higher adjusted ventilator liberation rate of 91.4% vs 56.0% in those without COVID-19. Functional ability was assessed with the change of Functional Status Score for the Intensive Care Unit (FSS-ICU) between admission and discharge. The adjusted mean change in FSS-ICU was significantly higher in the COVID-19 group than in the non-COVID-19 group: 9.49 (95% CI, 7.38-11.6) vs 2.08 (95% CI, 1.05-3.11), respectively (*P* < .001). Patients with COVID-19 experienced a shorter adjusted LOS at the LTACH with an adjusted hazard ratio of 1.57 (95% CI, 1.0-2.46; *P* = .05) compared with patients without COVID-19. We did not observe significant differences between the two groups regarding discharge location, but a trend toward need for lower level of care was found in patients with COVID-19.

**Interpretation:**

Our study suggests that patients with COVID-19 requiring MV and tracheostomy have a higher chance for recovery than those without COVID-19.

## Graphical Abstract

**Figure:**
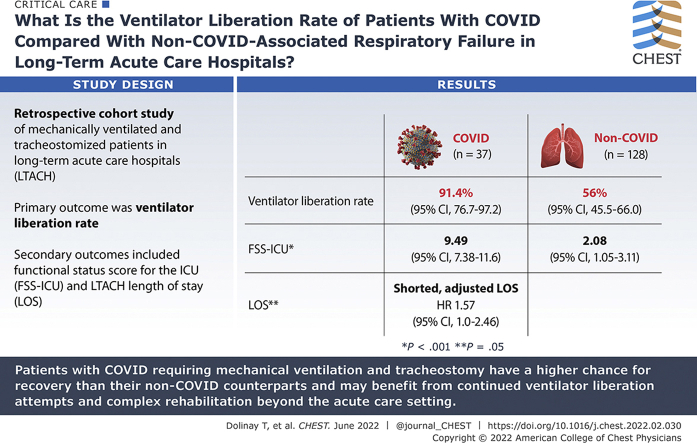

**Take-home Points**

**Research Question:** What is the ventilator liberation rate of patients who have received tracheostomy with COVID-19-associated respiratory failure compared with those with respiratory failure unrelated to COVID-19 in an long-term acute care hospital (LTACH)?

**Results:** Patients with COVID-19 achieved higher success for ventilator liberation and a higher level of functional recovery than those without COVID-19 on LTACH discharge.

**Interpretation:** Patients with COVID-19-associated respiratory failure may benefit from continued ventilator liberation attempts and complex rehabilitation beyond the acute care setting.

The COVID-19 pandemic has resulted in an unforeseen stress on health care systems. By January 2022, the United States alone saw 2.76 million COVID-19-related hospitalizations.^1^ It is estimated that in 10% of all hospitalized patients with COVID-19, severe acute respiratory failure develops requiring invasive mechanical ventilation (MV).^2^ Studies show that 8% to 13% of patients admitted to ICUs will undergo a tracheostomy to allow MV for extended periods.^3^ Tracheostomies usually are placed after 1 to 3 weeks of unsuccessful liberation from MV to facilitate longer-term recovery.^4^ They are well tolerated and improve ventilator outcomes,^5^^,^^6^ but the overall recovery potential of patients with COVID-19-associated respiratory failure who receive MV is unknown.

In the United States, after tracheostomy, medically stable patients who have a potential for liberation from MV often are transferred to long-term acute care hospitals (LTACHs) for continued care. LTACHs often are called “weaning facilities,” but they also provide rehabilitation for patients with chronic critical illness. Despite significant efforts by LTACHs, a multicenter analysis by Scheinhorn et al^7^ suggest that only about 54% of patients admitted for ventilator liberation are liberated successfully.

Our hypothesis was that patients admitted to the LTACH with COVID-19-associated respiratory failure have a better chance of achieving ventilator liberation and recovery than patients admitted for non-COVID-19-associated respiratory failure. To test our hypothesis, we studied 165 patients who were discharged during the COVID-19 pandemic from 2020 through 2021. In this retrospective cohort study, we analyzed the potential reasons for ventilator liberation success in this population with severe illness.

## Study Design and Methods

### LTACH Patient Population

Barlow Respiratory Hospital (BRH) is a 105-bed nonprofit LTACH in the Los Angeles area. BRH follows Centers for Medicare and Medicaid Services guidelines^8^ for admission (e-Table 1). BRH sees a generally sicker patient population (marked by a higher case mix index^9^) with a higher number of patients admitted for ventilator liberation and a higher number of admission denials than the national average (e-Table 2) based on data from the LTACH outcomes system (LTRAX).^10^

### Study Design and Patient Selection

The study was approved by the Western Institutional Review Board (Identifier: 1-1348082-1). To study the outcomes of MV at BRH during the COVID-19 pandemic from 2020 through 2021, we focused 242 consecutive discharges that occurred between June 1, 2020, and March 5, 2021. Study inclusion and exclusion criteria are listed in the online supplementary data. In brief, no difference was found in the admission criteria between patients with and without COVID-19. Patients with respiratory failure unrelated to COVID-19 were compared with patients with COVID-19-associated respiratory failure. Of the 242 patients, we excluded those who did not participate in our ventilator liberation program or harbored an oropharyngeal intubation tube on admission. The decision to exclude these patients from the ventilator liberation program was made by the admitting team and included the following criteria: (1) unresponsiveness (Glasgow coma scale score, < 6), (2) hemodynamic instability, (3) severe muscle weakness, (4) readmission with failed prior attempt at ventilator liberation, and (5) < 24-h stay at BRH. Patients with oropharyngeal tube intubation were excluded because their BRH hospital course was complicated by the need of airway procedures, including tracheostomy, which delayed ventilator liberation attempts. e-Table 3 presents patients who were excluded. Patients who participated in the ventilator liberation program received comprehensive respiratory, medical, physical, occupational, and speech therapy. Data were extracted from electronic medical records and were stored in a protected database.

### MV and Liberation

On admission, volume control, pressure control, and hybrid modes (eg, synchronized intermittent mandatory ventilation mode) were considered full support MV, and pressure support mode was considered spontaneous ventilator mode MV. Ventilator liberation was defined as cessation of MV for at least 48 h before discharge. Ventilator dependence was defined as continued MV on discharge.^11^

### Covariates and Comorbidities

To compare patients with and without COVID-19 adequately, we developed statistical models to correct for nine potential confounders (covariates) that have been described as modifiers of MV outcomes: (1) age,^12^ (2) gender,^13^ (3) race or ethnicity,^13^ (4) need for vasopressor^14^ at the short-term acute care hospital (STACH), (5) thrombocytopenia (platelet count < 150 × 10^3^/μL)^15^ on LTACH admission, (6) need for hemodialysis^16^ at LTACH, (7) length of stay (LOS)^17^ at the STACH, (8) the number of acute comorbidities, and (9) the number of chronic comorbidities^16^ (Table 1, e-Table 4). Comorbidities present before LTACH admission were reviewed from available medical records, and we generated a list of acute and chronic medical conditions that were considered for covariate adjustment. Acute comorbidities included: ARDS, sepsis, acute kidney injury, and acute VTE, including DVT and pulmonary embolism. Chronic stable comorbidities documented at the referring STACH included: hypertension, diabetes mellitus, congestive heart failure, prior cerebrovascular accident, coronary artery disease, obesity with BMI of > 30 kg/m^2^, and pulmonary fibrosis. The number of acute comorbidities of each patient was aggregated into a single number, and the number of chronic comorbidities was aggregated into a different number. Both were used as a covariate (Table 1). The definitions of comorbidities are shown in the online supplementary data.

**Table 1:** Patient Demographics With Comorbidities and Covariates

Demographic	Respiratory Failure Unrelated to COVID-19 Infection	COVID-19-Associated Respiratory Failure	*P* Value^a^
No. of patients	128	37	...
Age, y	69.3 ± 14.8	66.2 ± 12.2	.25
Male sex	80 (62.5)	29 (78.4)	.11
Race			.93
Asian	10 (7.8)	3 (8.1)	
Black	13 (10.2)	3 (8.1)	
White	95 (74.2)	31 (83.8)	
Pacific islander	3 (2.3)	0	
Unknown	7 (5.4)	0	
Ethnicity			.702
Hispanic	27 (21.1)	10 (27)	
Non-Hispanic	99 (77.3)	27 (73)	
Unknown	2 (1.6)	0	
Acute comorbidities			
ARDS	5 (3.9)	10 (27.0)	< .001
AKI	26 (20.3)	19 (51.4)	< .001
Sepsis	30 (23.4)	12 (32.4)	.372
Acute VTE	20 (15.6)	8 (21.6)	.544
Chronic comorbidities			
Diabetes	67 (52.2)	20 (54.1)	1
Hypertension	93 (72.7)	28 (75.7)	.877
CAD	31 (24.2)	8 (21.6)	.918
CHF	36 (28.1)	6 (16.2)	.211
CVA	40 (31.2)	6 (16.2)	.112
CKD	43 (33.6)	10 (27)	.58
BMI > 30 kg/m^2^	34 (26.8)	14 (37.8)	.273
Pulmonary fibrosis	4 (3.1)	2 (5.4)	.617
Covariates			
No. of acute comorbidities	0.5 ± 0.7	1.1 ± 1.0	< .001
No. of chronic comorbidities	2.9 ± 1.6	2.8 ± 1.5	.721
LOS at STACH, d	20.9 ± 15.4	35.2 ± 18.0	< .001
Vasopressor need at STACH	35 (27.3)	13 (35.1)	.475
Thrombocytopenia on LTACH admission	14 (10.9)	1 (2.7)	.194
Hemodialysis at LTACH	28 (21.9)	6 (16.2)	.604

Data are presented as No. (%) or mean ± SD, unless otherwise indicated. AKI = acute kidney injury; CAD = coronary artery disease; CHF = congestive heart failure; CKD = chronic kidney disease; CVA = cerebrovascular accident; LTACH = long-term acute care hospital; LOS = length of stay; STACH = short-term acute care hospital.

a Generated using univariate analysis comparing non-COVID-19 and COVID-19 groups. For categorical variables, the χ^2^ and Fisher exact tests were used. For continuous variables, the *t* test, nonparametric Wilcoxon rank-sum test, or log-rank test was used. *P* < .05 was considered significantly different.

### Functional Status Score for the Intensive Care Unit

To assess physical functional status, we used the Functional Status Score for the Intensive Care Unit (FSS-ICU).^18^ This score measures five basic abilities: (1) to roll, (2) to transfer from a lying to sitting position, (3) to sit at the edge of bed, (4) to transfer from sitting to standing, and (5) to walk. Each task is scored on a 0 (no function) to 7 (independent performance) scale. The minimum combined score is 0, and the maximum score is 35. The FSS-ICU has been validated in both the ICU^19^ and the LTACH^20^ setting. Patients were evaluated by a physical therapist on admission and discharge, and the change in FSS-ICU was used to assess for functional change.

### Discharge Disposition

We created five ordered discharge categories to show patient disposition. Discharges were ordered to represent more independent living as a better outcome: 1 = home, great; 2 = inpatient rehabilitation facility, good; 3 = skilled nursing facility, standard; 4 = STACH transfer, poor; and 5 = death, very poor.

### Statistical Analyses and Models

Statistical calculations were performed using R version 4.1.0 software (R Foundation for Statistical Computing). We worked with a complete database without missing values. Two-tailed *P* values were used, and *P* < .05 was considered statistically significant.

#### Univariate Analysis

Categorical variables between non-COVID-19 and COVID-19 groups were compared using the χ^2^ and Fisher exact tests. Continuous variables for demographics and outcomes were compared with *t* tests, nonparametric Wilcoxon rank-sum tests, or log-rank tests, as appropriate (Table 1).

#### Multivariate Analysis

**Figure 1:**
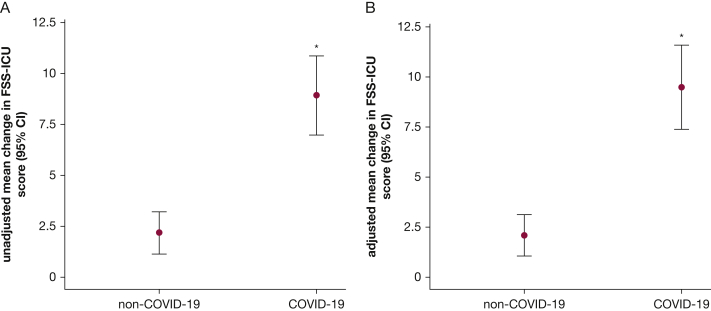
A, B, Graphs showing that patients with COVID-19 have better functional recovery. Physical function changes were assessed with FSS-ICU score on admission and discharge. Patients with COVID-19 showed a significantly higher difference in the mean change of the FSS-ICU score than peers without COVID-19. A, Unadjusted mean change in FSS-ICU score from admission to discharge in the group without COVID-19 was 2.18 (95% CI, 1.14-3.22) and 8.93 (95% CI, 7.0-10.88) in the group with COVID-19. The mean unadjusted difference in the mean change between the two groups was 6.76 (95% CI, 4.55-8.97; *P* < .001). B, Mean change after adjustment for covariates was 2.08 (95% CI, 1.05-3.11) in the group without COVID-19 and 9.49 (95% CI, 7.38-11.6) in the group with COVID-19. The adjusted mean difference in the mean change between the two groups was 7.41 (95% CI, 4.94-9.87; *P* < .001). Means and 95% CIs are shown on the dot plot. The statistical model was a linear regression one. ∗Significantly higher difference in the unadjusted and adjusted mean change of FSS-ICU score in patients with COVID-19. FSS-ICU = Functional Status Score for the Intensive Care Unit.

**Figure 2:**
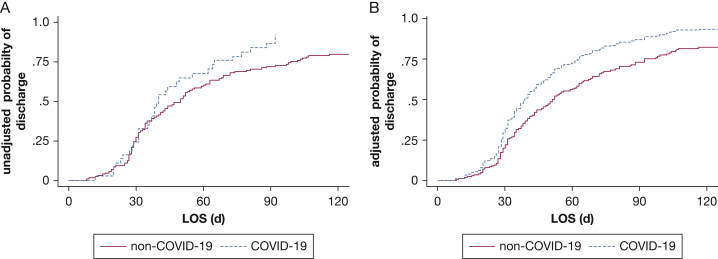
A, B, Graphs showing that patients with COVID-19 have a shorter long-term acute care hospital (LTACH) stay. Cumulative incidence curves were used to show the probability of discharge from LTACH in relationship to the LOS in days. Patients with COVID-19 (blue dotted line) showed a significantly longer LOS than their counterparts without COVID-19 (red solid line). A, Unadjusted hazard ratio (HR) of 1.29 (95% CI, 0.87-1.9; *P* = .2). B, Predicted HR after adjustment for covariates is shown: HR, 1.57 (95% CI, 1.0-2.46; *P* = .05). The statistical model was a Fine-Gray competing risk model with death as the competing risk. LOS = length of stay.

We used different statistical models to evaluate for each LTACH outcome. For all statistical models, unadjusted results comparing non-COVID-19 with COVID-19 groups were reported, along with results adjusted for the nine covariates listed above. First, the proportion of liberated vs dead or ventilator-dependent patients was assessed using a nominal logistic regression model. The unadjusted and adjusted proportions are reported (Table 2, e-Table 5). Second, the change in FSS-ICU between groups was assessed using linear regression (Fig 1, e-Table 6). Admission FSS-ICU was used as an additional covariate in this analysis in the adjusted model. Unadjusted and adjusted means and mean differences are reported. Third, LOS was assessed using a Fine-Gray competing risk model with death as the competing risk (Fig 2, e-Table 7). Unadjusted and adjusted hazard ratios and their 95% CIs is reported, and time to event curves are shown (Fig 1). Fourth, discharge disposition was assessed using an ordinal logistic model because the five discharge disposition categories were ordered (Table 3, e-Table 8). Model-based ORs and their 95% CIs, along with the corresponding unadjusted and model-adjusted proportions in each category are reported.

**Table 2:** Mechanical Ventilation Outcomes in Patients With and Without COVID-19

Outcome	Unadjusted for Covariates			Adjusted for Covariates		
	Without COVID-19	With COVID-19	Pairwise Unadjusted *P* Value^a^	Without COVID-19	With COVID-19	Pairwise Adjusted *P* Value^a^
Liberated	71 (55.5)	31 (83.8)	NA	56.0 (45.5-66.0)	91.4 (76.7-97.2)	NA
Ventilator dependent	43 (33.6)	3 (8.1)	.004	36.7 (27.3-47.2)	4.7 (1.1-17.3)	.001
Death	14 (10.9)	3 (8.1)	.289	7.3 (3.3-15.3)	3.9 (0.8-17.0)	.232

Data are presented as No. (%) or percentage (95% CI), unless otherwise indicated. NA = not applicable.

a Calculated using multinomial logistic regression. Pairwise *P* values represent the statistical difference in the number of patients who are ventilator dependent or dead compared with those liberated patients without COVID-19 vs with COVID-19. *P* values are shown for before and after adjustment for covariates. *P* < .05 was considered significantly different.

**Table 3:** Discharge Disposition of Patients Without and With COVID-19 Ordered by Location

Disposition	Unadjusted for Covariates			Adjusted for Covariates		
	Without COVID-19	With COVID-19	*P* Value^a^	Without COVID-19	With COVID-19	*P* Value^a^
Home	16 (12.5)	8 (21.6)	.017	8.7 (5.2-14.2)	17.9 (9.2-32.1)	.054
IRF	12 (9.4)	10 (27.0)		11.3 (7.3-17.5)	18.6 (11.3-29.3)	
SNF	68 (53.1)	12 (32.4)		57 (48.3-65.4)	52 (40.3-63.2)	
STACH transfer	18 (14.1)	4 (10.8)		14.3 (9.3-21.3)	7.5 (3.5-15.2)	
Death	14 (10.9)	3 (8.1)		8.6 (5.0-14.3)	3.9 (1.6-9.1)	

Data are presented as No. (%) or percentage (95% CI), unless otherwise indicated. Discharges were ordered in five categories and with the more independent living as the better outcome: 1 = home, great; 2 = IPR, good; 3 = SNF, standard; 4 = STACH transfer, poor; 5 = death, very poor. Unadjusted and adjusted proportions in each of the five discharge categories shown. IRF = inpatient rehabilitation facility; SNF = skilled nursing facility; STACH = short-term acute care hospital.

a Generated using an ordinal logistic regression model, with *P* < .05 representing a statistically significant number in discharge location for patients without COVID-19 vs with COVID-19.

## Results

### Demographics

Of 165 patients, 128 were admitted without COVID-19 and 37 (22.4%) were admitted with COVID-19 for ventilator liberation. Patient demographics are shown in Table 1 and in e-Table 4. The mean ± SD age was not significantly different between the non-COVID-19 and COVID-19 groups (69.3 ± 14.8 years vs 66.2 ± 12.2 years, respectively; *P* = .25). Both groups showed a male predominance (62.5% in the non-COVID-19 group and 78.4% in the COVID group; *P* = .11). The racial distribution was as follows: 74.2% White, 10.2% Black, 7.8% Asian, and 2.3% Pacific Islander in the non-COVID-19 group and 83.3% White, 8.1% Black, 8.1% Asian, and 0% Pacific Islander in the COVID-19 group. The non-COVID-19 group was 21.1% Hispanic and the COVID-19 group was 27% Hispanic. No difference was found in the type of ventilation (full support vs spontaneous) between the non-COVID-19 and COVID-19 groups on admission (86.7% vs 95% with full support; *P* = .34) (e-Table 4).

### Comorbidity and Covariate Analysis

We first compared the percentage of comorbidities between patients without COVID-19 and those with COVID-19 (Table 1). Of comorbid conditions, the presence of ARDS (27% vs 3.9%, respectively; *P* < .001) and acute kidney injury (51.4% vs 20.3%, respectively; *P* < .001) on admission was more common in the COVID-19 group than in the non-COVID-19 group. Other comorbidities did not differ significantly between groups. However, the mean ± SD number of acute comorbidities was higher in the COVID-19 group than in the non-COVID-19 group: 1.1 ± 0.7 vs 0.5 ± 1 (*P* < .001). Patients in the COVID-19 group spent significantly longer in the STACH than patients in the non-COVID-19 group before LTACH transfer. Median stay was 35.0 days (interquartile range [IQR], 21.0-43.0 days) for patients with COVID-19 vs 16.5 days (IQR, 10.0-27.0 days) for patients without COVID-19 (*P* < .001). No difference was found in the amount of vasopressor need at the STACH, in the presence of thrombocytopenia on LTACH admission, or in the need for hemodialysis at the LTACH between the two groups. These data suggest that patients with COVID-19 spend longer at the STACH and have more acute illness on LTACH admission, but are not significantly sicker than those without COVID-19 at the time of LTACH transfer.

### Ventilator Liberation

The primary outcome was MV liberation. To evaluate the success of MV liberation on discharge, we considered three mutually exclusive outcomes: (1) liberated from positive pressure ventilation, (2) ventilator dependent, and (3) died during hospitalization. We compared the proportion of patients who achieved ventilator liberation, who remained ventilator dependent, or who died as inpatients between the non-COVID-19 and COVID-19 groups (Table 2). The unadjusted proportion of ventilator-liberated patients was 55.5% in the non-COVID-19 group and 83.8% in the COVID-19 group. After adjustment for covariates, success of ventilator liberation was 56.0% (95% CI, 45.5%-66.0%) in the non-COVID-19 group and 91.4% (95% CI, 76.7%-97.2%) in the COVID-19 group. The unadjusted proportion of patients with ongoing ventilator support at discharge was 33.6% in the non-COVID-19 group and 8.1% in the COVID-19 group (*P* = .004). The adjusted proportion was 36.7% (95% CI, 27.3%-47.2%) and 4.7% (95% CI, 1.1%-17.3%), respectively (*P* = .001). No significant difference was found in the unadjusted (10.9% vs 8.1%; *P* = .289) and adjusted inpatient mortality between groups: 7.3% (95% CI, 3.3%-15.3%) vs 3.9% (95% CI, 0.8%-17.0%; *P* = .232). These data show that patients with COVID-19 were more likely to be liberated from MV than those without COVID-19.

### Functional Status

Admission FSS-ICUs were low and not significantly different between non-COVID-19 and COVID-19 groups (mean ± SD, 2.7 ± 3.1 vs 1.9 ± 1.7; *P* = .135) (e-Table 4). Low FSS-ICUs signify a severely disabled population on admission. To evaluate for functional recovery, we compared mean changes between admission and discharge for the two groups (Fig 1). Unadjusted changes are shown in Figure 2A: 2.18 (95% CI, 1.14-3.22) in the non-COVID-19 group vs 8.93 (95% CI, 7.0-10.88) in the COVID-19 group (*P* < .001). Adjusted changes are depicted in Figure 2B: 2.08 (95% CI, 1.05-3.11) in the non-COVID-19 group and 9.49 (95% CI, 7.38-11.6) in the COVID-19 group (*P* < .001). The adjusted mean difference in the mean change was 7.41 (95% CI, 4.94-9.87; *P* < .001). These results suggest that patients with COVID-19 were able to achieve a higher level of functional recovery during LTACH stay.

### LOS

The median LOS to discharge from LTACH was longer for patients without COVID-19 compared with patients with COVID-19, with an unadjusted median of 50 days (IQR, 30-99 days) vs 40 days (IQR, 31-65 days) and an adjusted median of 52 days (IQR, 31-97 days) vs 39 days (IQR, 28-63 days), respectively (e-Table 7). Figure 2A shows the cumulative incidence curves for the unadjusted probability of discharge in relationship to LOS, and Figure 2B shows the curves after adjustment for covariates. The unadjusted hazard ratio for LOS in the COVID-19 group vs the non-COVID-19 group was 1.29 (95% CI, 0.87-1.9; *P* = .2), and the corresponding adjusted hazard ratio was 1.57 (95% CI, 1.00-2.46; *P* = .05). These results indicate that discharge was significantly faster in the COVID-19 group.

### Discharge Disposition

To evaluate outcomes further, we compared the discharge disposition between the two groups. The unadjusted OR for discharge to more independent living for patients without COVID-19 vs patients with COVID-19 was 0.43 (95% CI, 0.22-0.86; *P* = .017), and after adjustment for covariates, the OR was 0.43 (95% CI, 0.19-1.0; *P* = .054). ORs and *P* values are listed from the statistical model in e-Table 8. Table 3 shows the unadjusted and adjusted proportions in each of the five discharge categories. Although we did not observe statistically significant differences between the two groups in any of any discharge categories after adjustment for covariates, our results suggest that patients with COVID-19 likely needed a lower level of care after discharge than the patients without COVID-19.

## Discussion

MV is one of the greatest inventions of modern medicine, and it is the foundation of ICU care. It is estimated that 2.8% of all hospital admissions require invasive MV in the United States,^21^ and 39% of ICU patients undergo MV via endotracheal tubes.^22^ Although MV originally was intended for short-time life support, approximately 5% of patients require prolonged MV beyond 21 days.^23^ Tracheostomies increasingly are performed for patients requiring prolonged MV,^5^ and patients who have received tracheostomy often are transferred to long-term care facilities for recovery and for potential ventilator liberation.^24^ Despite advances in the care of patients who received MV, the outcomes are poor and only about 50% to 60% of patients achieve liberation from MV.^7^^,^^11^

The COVID-19 pandemic has resulted in a major rethinking of the way we practice and apply MV. Patients with severe acute respiratory failure resulting from COVID-19 sought treatment at hospitals in unprecedented numbers. Mechanical ventilators became scarce and highly wanted resources to improve survival.^25^ Simultaneously, concerns about staff exposure to SARS-CoV-2 and poor outcomes of patients who receive MV hindered initiation of MV in patients with infection.^26^ For patients who receive MV, further delays are caused by safety concerns regarding performing tracheostomies in patients with coronavirus infection.^27^

The current focus in COVID-19 response is the acute hospital system. Little is known regarding what happens to those who survived the acute phase of the disease with chronic critical illness. To our knowledge, our study is the first to report outcomes of patients with COVID-19 requiring MV beyond acute care hospitalization. We believe that studying this population is of critical importance because they represent a significant burden on the health care system. Their rehabilitation success also may provide an examples of how to care for the growing number of patients with chronic critical illness.

In our study, we described a severely disabled patient population signified by low admission FSS-ICU. ARDS and acute kidney injury were more common in the COVID-19 group, which is consistent with the findings of Piroth et al.^2^ After adjustments for covariates, patients with COVID-19 showed an increased rate of ventilator liberation, better physical recovery, and shorter LTACH LOS. The most important finding is that 83.8% (91.4% after adjustment for covariates) of patients with COVID-19 achieved liberation from MV, which is significantly higher than the usual liberation rate at LTACHs.^7^^,^^11^ Although no significant difference was found in overall discharge disposition, a trend toward patients with COVID-19 requiring a lower level of care than those without COVID-19 was found. In-hospital mortality remained low in both groups.

Based on our results, we speculate that the higher rate of ventilator liberation success for those with COVID-19 is related to their better recovery potential. First, patients with COVID-19 spent more time at a STACH and already may have started to recover at the time of LTACH admission. Second, they had a significantly higher number of acute comorbidities before LTACH admission than patients without COVID-19, but shock (measured by vasopressor use), thrombocytopenia, and hemodialysis were not different between the groups. As acute illnesses improved, patients with COVID-19 may have recovered faster. This may be particularly important in the case of patients requiring hemodialysis because prolonged hemodialysis need is associated with poor hospital outcomes.^28^ We believe that patients with COVID-19 represent a unique population in the post-acute-care setting. Allowing time for rehabilitation and ventilator liberation attempts can help them to achieve a recovery beyond what is seen in the general LTACH population.

Interestingly, despite the better recovery potential of patients with COVID-19, no significant difference was found in the discharge disposition of patients. We believe our results are confounded by the difficulty of taking care of patients with multiple medical needs. Many patients require long-term tracheostomies and continued oxygen supply, which is difficult to arrange at home, even if the patient otherwise is independent. Acute inpatient rehabilitation facilities have limited capability of taking care of patients with feeding tubes, decubitus ulcers, and hemodialysis, which may necessitate a lower level of care at a skilled nursing facility. Furthermore, in the United States, medical insurance status often is a determinant of placement in skilled nursing facilities, even for patients, who have a good rehabilitation potential.

Our study has several strengths. First, it provides new insight into the recovery of patients with COVID-19. Second, it confirms previous findings that patients who undergo prolonged MV represent a heterogeneous group with various level of recovery potential. Third, it emphasizes the importance of rehabilitation assessment and complex rehabilitation in the LTACH setting.

Our study also has several limitations. First, patient selection may have resulted in the elimination of potential candidates for ventilator liberation. In our retrospective cohort study, we enrolled small group of severely disabled, tracheostomized, and ventilated patients at a single institution in Los Angeles. Patient enrollment in the ventilator liberation program was decided by the admitting team of specialists based on medical history and physical examination findings (e-Table 3). BRH specializes in patients who receive MV and shows higher ventilator liberation success than the national average, but also rejects more patients for admission than the national average (e-Tables 1, 2). However, we tried to limit the effect of patient selection in our study by applying the same enrollment criteria to both groups and used covariates to control for potential confounders (Table 1, e-Table 4). Second, although no difference was found in the number of chronic comorbidities or in the initial ventilator settings on LTACH admission, our analysis does not control adequately for disease severity. It is possible that patients without COVID-19 harbored more severe underlying diseases with lesser chance of improvement. Third, delays in tracheostomy can alter the course of MV and can contribute to prolonged hospitalization.^29^ For this reason, we included in our analysis patients who arrived at the LTACH with a tracheostomy. However, we cannot exclude that the decision to perform tracheostomy and when to perform the tracheotomy at the STACH may have been biased by COVID-19 status. Fourth, we were unable to assess the long-term outcomes of patients beyond LTACH care, which would provide additional insight into their recovery. Fifth, our database is limited to the admission records available to the study team. Despite adjustment for covariates, a possibility exists for unmeasured covariates, including the choice of COVID-19-specific medications, which could have influenced outcomes.

## Interpretation

Our study provides new evidence that patients with COVID-19-associated respiratory failure requiring MV via tracheostomy have a better recovery potential than those without COVID-19, marked by improved ventilator liberation, better physical functioning, and shorter LTACH stay.

## Supplementary Data

e-Online Data
