# Supplementary material for: Mechanical Ventilator Liberation of Patients With COVID-19 in Long-term Acute Care Hospital
Source: Chest. 2022 Feb 25;161(6):1517–25. doi: 10.1016/j.chest.2022.02.030 (PMC8875856; doi:10.1016/j.chest.2022.02.030)
Supplement: e-Online Data [file mmc1.docx]

**Online supplement**

**Mechanical ventilator liberation of COVID-19 patients in long-term acute care hospital**

**Running head:** Ventilator liberation in LTACH

^1,2^Tamas Dolinay MD, PhD, ^1,2^Dale Jun MD,^1^Lucia Chen MS, ^1^Jeffrey Gornbein DrPH

^1^University of California Los Angeles Department of Medicine and ^2^Barlow Respiratory Hospital

Study inclusion criteria:

1. Patients admitted to Barlow Respiratory Hospital (BRH) for acute or acute on chronic respiratory failure requiring invasive positive pressure mechanical ventilation via tracheostomy

Study exclusion criteria:

1. Patients with hemodynamic instability on admission
2. Patients requiring vasopressor medication to stabilize blood pressure
3. Systolic blood pressure less than 90mmHg
4. Pulse less than 50 or greater than 130 beats per minute or change by more than 20 from baseline
5. Respiratory rate greater than 35/min
6. Oxygen saturation less than 90%
7. Body temperature greater than 38C
8. Prominent accessory muscle use
9. Spontaneous lung tidal volume less than 250ml representing severe muscle weakness
10. Oropharyngeal tube intubation in place at the time of BRH admission
11. Coma, measured by Glasgow Coma Scale (GCS) less than 6 on admission
12. At least one previous admission to BRH with unsuccessful ventilator liberation attempt
13. Length of Stay (LOS) at BRH less than 24hours, which prevented continued assessment

Definitions of comorbidities:

Acute kidney injury (AKI): 2-fold increase in serum creatinine or 50% decrease in glomerular filtration rate within 48 hours^1^.

Acute respiratory distress syndrome (ARDS): New bilateral pulmonary infiltrates on chest radiography within the 7 days that is not related to pulmonary edema. The ratio of partial arterial oxygen pressure and fractional inspired oxygen is less than 300 measured in the presence of at least 5 cmH_2_O continuous positive airway pressure (CPAP) ventilation or positive end expiratory pressure (PEEP)^2^.

Sepsis: Defined as life-threatening organ dysfunction caused by dysregulated host response to infection^3^ with or without hypotension during hospital stay.

Acute venous thromboembolism (VTE): Acute deep venous thrombosis and/or pulmonary embolism^4^ during hospital stay.

Chronic comorbidities: chronic kidney disease (CKD), congestive heart failure (CHF), diabetes mellitus (DM), coronary artery disease (CAD), prior cerebrovascular incident (CVA), obesity (body mass index>30), hypertension and pulmonary fibrosis that were stable on admission to Barlow Respiratory Hospital.

References

1. Bellomo R, Ronco C, Kellum JA, Mehta RL, Palevsky P, Acute Dialysis Quality Initiative w. Acute renal failure - definition, outcome measures, animal models, fluid therapy and information technology needs: the Second International Consensus Conference of the Acute Dialysis Quality Initiative (ADQI) Group. *Crit Care.* 2004;8(4):R204-212.

2. Force ADT, Ranieri VM, Rubenfeld GD, et al. Acute respiratory distress syndrome: the Berlin Definition. *JAMA.* 2012;307(23):2526-2533.

3. Singer M, Deutschman CS, Seymour CW, et al. The Third International Consensus Definitions for Sepsis and Septic Shock (Sepsis-3). *JAMA.* 2016;315(8):801-810.

4. Beckman MG, Hooper WC, Critchley SE, Ortel TL. Venous thromboembolism: a public health concern. *Am J Prev Med.* 2010;38(4 Suppl):S495-501.

e-Table 1. Admission criteria to Barlow Respiratory Hospital

| **Respiratory care** |
| --- |
| ventilator liberation |
| acute respiratory failure |
| acute respiratory distress syndrome |
| pulmonary fibrosis |
| complex pneumonia |
| chronic obstructive pulmonary disease |
| **Medically complex care management** |
| renal failure/hemodialysis |
| congestive heart failure |
| immunosuppressed patient care |
| pre- and post-organ transplant care |
| multiple organ failure |
| post traumatic injury |
| surgical complications |
| infectious diseases |
| sepsis and septicemia |
| **Wound care** |
| complex wounds osteomyelitis |
| fistulas/abscesses |
| pressure injuries |
| post-surgical wounds |
| necrotic conditions |

e-Table 2. Barlow Respiratory Hospital (BRH) 2019 ventilator outcomes compared to national statistics

|  | BRH | national |
| --- | --- | --- |
| case mix index | 1.35 | 1.19 |
| admissions | 918 | 124,467 |
| admission denials (% of total applicants) | 1002 (52) | 32,346 (20) |
| invasive ventilation admissions (% of admissions) | 405 (44) | 28,106 (22.5) |
| invasive ventilation admissions for liberation (% of admissions) | 293 (31.9) | 24,938 (20) |
| ventilator liberation rate (% admissions for liberation) | 177 (60.4) | 12,942 (51.9) |

e-Table 3. Patient study population and decision on exclusion from ventilator liberation program

|  | total (N)=242 | COVID (N)=58 | non-COVID (N)=184 |
| --- | --- | --- | --- |
| enrolled patients | 165 | 37 | 128 |
| excluded patients (% of total patients) | 77 (31.8) | 21 (36.2) | 56 (30.4) |
| **exclusion (% of total patients)** |  |  |  |
| hemodynamic instability | 4 (1.6) | 2 (3.4) | 2 (1.5) |
| oropharyngeal tube present on admission | 5 (2) | 1 (1.7) | 4 (2.2) |
| severe muscle weakness | 7 (2.9) | 1 (1.7) | 6 (3.2) |
| unresponsive on admission, GCS<6 | 6 (2.5) | 0 (0) | 6 (3.2) |
| readmissions with multiple failed liberation attempts | 45 (18.6) | 10 (17.2) | 35 (19) |
| LOS<24 hrs | 10 (4.1) | 7 (12) | 3 (1.6) |

Abbreviations: GCS=Glasgow Coma Scale, LOS=length of stay

e-Table 4 Stratified patient data

|  |  | Non-COVID | COVID | *P*-value |
| --- | --- | --- | --- | --- |
|  |  | (N=128) | (N=37) |  |
| **Demographics** | |  |  |  |
| Age |  |  |  | 0.25 |
|  | Mean (SD) | 69.3 (14.8) | 66.2 (12.2) |  |
|  | Median (Q1-Q3) | 71.0 (61.0-79.0) | 67.0 (60.0-75.0) |  |
|  | Min-Max | 28.0-104.0 | 27.0-87.0 |  |
| Sex |  |  |  | 0.11 |
|  | Male | 80 (62.5%) | 29 (78.4%) |  |
|  | Female | 48 (37.5%) | 8 (21.6%) |  |
| Ethnicity | |  |  | 0.7 |
|  | Hispanic | 27 (21.1%) | 10 (27.0%) |  |
|  | Non-Hispanic | 99 (77.3%) | 27 (73.0%) |  |
|  | Unknown | 2 (1.6%) | 0 (0.0%) |  |
| Race | |  |  | 0.93 |
|  | Asian | 10 (7.8%) | 3 (8.1%) |  |
|  | Black or African American | 13 (10.2%) | 3 (8.1%) |  |
|  | White | 95 (74.2%) | 31 (83.8%) |  |
|  | Native Hawaiian/Pacific Islander | 3 (2.3%) | 0 (0.0%) |  |
|  | Other Race | 3 (2.3%) | 0 (0.0%) |  |
|  | Unknown | 4 (3.1%) | 0 (0.0%) |  |
|  |  |  |  |  |
| **Admission Characteristics and Outcomes** | |  |  |  |
| LOS at STACH | |  |  | <0.001 |
|  | Mean (SD) | 20.9 (15.4) | 35.2 (18.0) |  |
|  | Median (Q1-Q3) | 16.5 (10.0-27.0) | 35.0 (21.0-43.0) |  |
|  | Min-Max | 1.0-89.0 | 2.0-78.0 |  |
| Type of invasive ventilator support on LTACH admission (%) | |  |  | 0.34 |
|  | full support | 111 (86.7) | 35 (95) |  |
|  | spontaneous mode | 17 (13.3) | 2 (5) |  |
| Days to liberate from ventilator | |  |  | 0.002 |
|  | Mean (SD) | 21.1 (36.3) | 21.3 (18.1) |  |
|  | Median (Q1-Q3) | 11.0 (7.0-21.0) | 16.0 (12.0-22.5) |  |
|  | Min-Max | 4.0-261.0 | 4.3-79.0 |  |
| Airway at discharge (%) | |  |  | <0.001 |
|  | Capped tracheostomy | 11 (8.6%) | 0 (0.0%) |  |
|  | Endotracheal | 0 (0.0%) | 0 (0.0%) |  |
|  | Montgomery Button | 1 (0.8%) | 2 (5.4%) |  |
|  | Natural | 5 (3.9%) | 12 (32.4%) |  |
|  | Tracheostomy | 111 (86.7%) | 23 (62.2%) |  |
| Ventilator Days | |  |  | 0.01 |
|  | Mean (SD) | 37.4 (46.9) | 19.5 (13.7) |  |
|  | Median (Q1-Q3) | 22.5 (9.0-44.2) | 16.0 (12.0-22.0) |  |
|  | Min-Max | 4.0-271.0 | 4.0-72.0 |  |
| FSS at Admission | |  |  | 0.135 |
|  | Mean (SD) | 2.7 (3.1) | 1.9 (1.7) |  |
|  | Median (Q1-Q3) | 1.0 (1.0-3.2) | 1.0 (1.0-2.0) |  |
|  | Min-Max | 0.0-16.0 | 0.0-6.0 |  |
| FSS at Discharge | |  |  | <0.001 |
|  | Mean (SD) | 4.9 (6.5) | 10.7 (8.3) |  |
|  | Median (Q1-Q3) | 1.0 (1.0-9.2) | 12.0 (1.0-18.0) |  |
|  | Min-Max | 0.0-26.0 | 0.0-24.0 |  |
| Hemodialysis at LTACH | |  |  | 0.604 |
|  | Yes | 28 (21.9%) | 6 (16.2%) |  |
|  | No | 100 (78.1%) | 31 (83.8%) |  |
| Need for Vasopressors at STACH (%) | |  |  | 0.475 |
|  | Yes | 35 (27.3%) | 13 (35.1%) |  |
|  | No | 93 (72.7%) | 24 (64.9%) |  |
| Thrombocytopenia at LTACH admission (%) | |  |  | 0.194 |
|  | Yes | 14 (10.9%) | 1 (2.7%) |  |
|  | No | 114 (89.1%) | 36 (97.3%) |  |
| **Comorbidities** | |  |  |  |
| ARDS (%) | |  |  | <0.001 |
|  | Yes | 5 (3.9%) | 10 (27.0%) |  |
|  | No | 123 (96.1%) | 27 (73.0%) |  |
| sepsis (%) | |  |  | 0.372 |
|  | Yes | 30 (23.4%) | 12 (32.4%) |  |
|  | No | 98 (76.6%) | 25 (67.6%) |  |
| AKI (%) | |  |  | <0.001 |
|  | Yes | 26 (20.3%) | 19 (51.4%) |  |
|  | No | 102 (79.7%) | 18 (48.6%) |  |
| acute VTE (%) | |  |  | 0.544 |
|  | Yes | 20 (15.6%) | 8 (21.6%) |  |
|  | No | 108 (84.4%) | 29 (78.4%) |  |
| CKD (%) | |  |  | 0.58 |
|  | Yes | 43 (33.6%) | 10 (27.0%) |  |
|  | No | 85 (66.4%) | 27 (73.0%) |  |
| hypertension (%) | |  |  | 0.877 |
|  | Yes | 93 (72.7%) | 28 (75.7%) |  |
|  | No | 35 (27.3%) | 9 (24.3%) |  |
| diabetes mellitus (%0 | |  |  | 1 |
|  | Yes | 67 (52.3%) | 20 (54.1%) |  |
|  | No | 61 (47.7%) | 17 (45.9%) |  |
| CHF (%) | |  |  | 0.211 |
|  | Yes | 36 (28.1%) | 6 (16.2%) |  |
|  | No | 92 (71.9%) | 31 (83.8%) |  |
| CAD (%) |  |  |  | 0.914 |
|  | Yes | 31 (24.2%) | 8 (21.6%) |  |
|  | No | 97 (75.8%) | 29 (78.4%) |  |
| CVA (%) |  |  |  | 0.112 |
|  | Yes | 40 (31.2%) | 6 (16.2%) |  |
|  | No | 88 (68.8%) | 31 (83.8%) |  |
| Obesity (%) | |  |  | 0.273 |
|  | Yes | 34 (26.8%) | 14 (37.8%) |  |
|  | No | 94 (73.4%) | 23 (62.2%) |  |
| Pulmonary Fibrosis (%0 | |  |  | 0.617 |
|  | Yes | 4 (3.1%) | 2 (5.4%) |  |
|  | No | 124 (96.9%) | 35 (94.6%) |  |
| Number of Acute Comorbidities | |  |  | <0.001 |
|  | Mean (SD) | 0.5 (0.7) | 1.1 (1.0) |  |
|  | Median (Q1-Q3) | 0.0 (0.0-1.0) | 1.0 (0.0-2.0) |  |
| Number of Chronic Comorbidities | |  |  | 0.721 |
|  | Mean (SD) | 2.9 (1.6) | 2.8 (1.5) |  |
|  | Median (Q1-Q3) | 3.0 (2.0-4.0) | 3.0 (2.0-4.0) |  |
|  | Min-Max | 0.0-6.0 | 0.0-7.0 |  |

Abbreviations: COVID=COVID-19-associated respiratory failure, non-COVID= non- COVID-19-associated respiratory failure, LOS=length of stay, SD=standard deviation, Q=Quartile, AKI=acute kidney injury, ARDS=acute respiratory distress syndrome, DVT=deep vein thrombosis, CAD=coronary artery disease, CKD= chronic kidney disease, CVA= cerebrovascular accident, BMI=body mass index, HD= hemodialysis, LTACH= long-term acute care hospital, STACH=short-term acute care hospital, FSS-ICU=Functional Status Score for the Intensive Care Unit. The same abbreviations are used in subsequent tables.

e-Table 5 Statistical model for ventilator liberation

OR=odds ratio, CI=confidence interval. The same abbreviations are used in subsequent tables.

| Method: Multinomial logistic regression | |  |
| --- | --- | --- |
| **Unadjusted results** |  |  |
|  | OR (95% CI) | *P*-value |
| COVID vs. non-COVID |  |  |
| Died vs. Liberated | 0.49 (0.13, 1.83) | 0.29 |
| Vent Dep vs. Liberated | 0.16 (0.05, 0.55) | 0.004 |
| **Adjusted results** |  |  |
| COVID vs. non-COVID |  |  |
| Died vs. Liberated | 0.33 (0.05, 2.04) | 0.232 |
| Vent Dep vs. Liberated | 0.08 (0.02, 0.38) | 0.001 |

| e-Table 6. Statistical model for change in Functional Status Score for the Intensive Care Unit (FSS-ICU) score   \| Method: Linear regression \|  \|  \| \| --- \| --- \| --- \| \| **Unadjusted results** \|  \|  \| \|  \| Estimate (95% CI) \| *P*-value \| \| COVID vs. non-COVID \| 6.76 (4.55, 8.97) \| <0.001 \| \| FSS at Admit \| 0.25 (-0.08, 0.57) \| 0.14 \| \| **Adjusted results** \|  \|  \| \|  \| Estimate (95% CI) \| P-value \| \| COVID vs. non-COVID \| 7.41 (4.94, 9.87) \| <0.001 \| \| Age \| 0 (-0.07, 0.08) \| 0.91 \| \| Male \| -0.42 (-2.43, 1.59) \| 0.68 \| \| Hemodialysis at LTACH \| -3.08 (-5.44, -0.73) \| 0.011 \| \| Asian vs. Non-Hispanic White \| -1.2 (-4.69, 2.3) \| 0.5 \| \| Black vs. Non-Hispanic White \| 1.14 (-2.11, 4.39) \| 0.49 \| \| Hispanic vs. Non-Hispanic White \| 2.04 (-0.22, 4.3) \| 0.08 \| \| Other/Unknown vs. White \| 6.31 (1.58, 11.04) \| 0.01 \| \| Number of Acute Comorbidities \| -1.94 (-3.35, -0.53) \| 0.008 \| \| Number of Chronic Comorbidities \| -0.17 (-0.82, 0.47) \| 0.6 \| \| Vasopressors at STACH \| 0.95 (-1.25, 3.15) \| 0.4 \| \| Thrombocytopenia at LTACH Admission \| 0.7 (-2.55, 3.95) \| 0.67 \| \| LOS at STACH \| 0.05 (-0.01, 0.1) \| 0.11 \| \| FSS at Admit \| 0.21 (-0.14, 0.56) \| 0.24 \| |  |  |
| --- | --- | --- | --- | --- | --- | --- | --- | --- | --- | --- | --- | --- | --- | --- | --- | --- | --- | --- | --- | --- | --- | --- | --- | --- | --- | --- | --- | --- | --- | --- | --- | --- | --- | --- | --- | --- | --- | --- | --- | --- | --- | --- | --- | --- | --- | --- | --- | --- | --- | --- | --- | --- | --- | --- | --- | --- | --- | --- | --- | --- | --- | --- | --- | --- | --- |
|  | |  |
| \|  \|  \| \|  \| \| --- \| --- \| --- \| --- \| \|  \|  \| \|  \| \|  \|  \| \|  \| \|  \|  \| \|  \| \|  \|  \| \|  \| \|  \|  \| \|  \| \|  \|  \| \|  \| \|  \|  \| \|  \| \|  \|  \| \|  \| \|  \|  \| \|  \| \|  \|  \| \|  \|  \| \|  \| \|  \|  \| \|  \| \|  \|  \| \|  \| |  |  |
|  |  |  |
| e-Table 7. Statistical model for length of stay (LOS) at LTACH  HR=hazard ratio, IQR=interquartile range   \| Method: Fine-Gray competing risk model with death as competing risk \| \|  \| \| --- \| --- \| --- \| \| **Unadjusted results** \|  \|  \| \|  \| Subdistribution HR (95% CI) \| *P*-value \| \| COVID vs. non-COVID \| 1.29 (0.87, 1.90) \| 0.2 \| \| \| Unadjusted median LOS (IQR):  Non-COVID: 50 (30-99) \| \| \| --- \| --- \| \| COVID: 40 (31-65) \| \| \|  \|  \| \|  \|  \| \| **Adjusted results** \|  \|  \| \|  \| Subdistribution HR (95% CI) \| P-value \| \| COVID vs. non-COVID \| 1.57 (1, 2.46) \| 0.05 \| \| Age \| 0.99 (0.98, 1.01) \| 0.38 \| \| Male \| 1.03 (0.74, 1.43) \| 0.86 \| \| Hemodialysis at LTACH \| 0.39 (0.24, 0.65) \| <0.001 \| \| Asian vs. Non-Hispanic White \| 1 (0.65, 1.55) \| 0.99 \| \| Black vs. Non-Hispanic White \| 1.25 (0.74, 2.11) \| 0.4 \| \| Hispanic vs. Non-Hispanic White \| 1.24 (0.83, 1.86) \| 0.29 \| \| Other/Unknown vs. White \| 1.59 (0.82, 3.06) \| 0.17 \| \| Number of Acute Comorbidities \| 0.78 (0.6, 1.01) \| 0.057 \| \| Number of Chronic Comorbidities \| 0.99 (0.88, 1.11) \| 0.84 \| \| Vasopressors at STACH \| 1.3 (0.85, 2) \| 0.22 \| \| Thrombocytopenia at LTACH admission \| 0.98 (0.56, 1.73) \| 0.95 \| \| LOS at STACH \| 0.99 (0.98, 1) \| 0.084 \| \| \| Adjusted mean LOS (IQR):  Non-COVID: 52 (31-97) \| \| \| --- \| --- \| \| COVID: 39 (28-63) \| \| \|  \|  \| \|  \|  \| |  |  |
|  |  |  |

e-Table 8. Statistical model for discharge disposition

| Method: Ordinal logistic regression | |  |
| --- | --- | --- |
| **Unadjusted results** |  |  |
|  | Proportional OR (95% CI) | *P*-value |
| COVID vs. non-COVID | 0.43 (0.22, 0.86) | 0.017 |
| **Adjusted results** |  |  |
|  | Proportional OR (95% CI) | P-value |
| COVID vs. non-COVID | 0.43 (0.19, 1.01) | 0.054 |
| Age | 1 (0.98, 1.03) | 0.89 |
| Male | 1.06 (0.55, 2.05) | 0.86 |
| Hemodialysis at LTACH | 6.3 (2.66, 14.93) | <0.001 |
| Asian vs. Non-Hispanic White | 0.86 (0.26, 2.78) | 0.8 |
| Black vs. Non-Hispanic White | 0.32 (0.11, 0.92) | 0.035 |
| Hispanic vs. Non-Hispanic White | 0.6 (0.28, 1.27) | 0.18 |
| Other/Unknown vs. White | 0.23 (0.05, 1.04) | 0.056 |
| Number of Acute Comorbidities | 1.21 (0.74, 1.97) | 0.45 |
| Number of Chronic Comorbidities | 1.12 (0.9, 1.38) | 0.31 |
| Vasopressors at STACH | 0.57 (0.27, 1.2) | 0.14 |
| Thrombocytopenia at LTACH admission | 0.88 (0.29, 2.69) | 0.82 |
| LOS at STACH | 0.98 (0.96, 1) | 0.13 |
